# Machine-Learning-Based Bibliometric Analysis of Pancreatic Cancer Research Over the Past 25 Years

**DOI:** 10.3389/fonc.2022.832385

**Published:** 2022-03-28

**Authors:** Kangtao Wang, Ingrid Herr

**Affiliations:** Molecular OncoSurgery, Section Surgical Research, Department of General, Visceral and Transplantation Surgery, University of Heidelberg, Heidelberg, Germany

**Keywords:** machine learning, pancreatic ductal adenocarcinoma, natural language processing (NLP), latent Dirichlet allocation (LDA) algorithm, bibliometric (R-package), Python (programming language)

## Abstract

Machine learning and semantic analysis are computer-based methods to evaluate complex relationships and predict future perspectives. We used these technologies to define recent, current and future topics in pancreatic cancer research. Publications indexed under the Medical Subject Headings (MeSH) term ‘Pancreatic Neoplasms’ from January 1996 to October 2021 were downloaded from PubMed. Using the statistical computing language R and the interpreted, high-level, general-purpose programming language Python, we extracted publication dates, geographic information, and abstracts from each publication’s metadata for bibliometric analyses. The generative statistical algorithm “latent Dirichlet allocation” (LDA) was applied to identify specific research topics and trends. The unsupervised “Louvain algorithm” was used to establish a network to identify relationships between single topics. A total of 60,296 publications were identified and analyzed. The publications were derived from 133 countries, mostly from the Northern Hemisphere. For the term “pancreatic cancer research”, 12,058 MeSH terms appeared 1,395,060 times. Among them, we identified the four main topics “Clinical Manifestation and Diagnosis”, “Review and Management”, “Treatment Studies”, and “Basic Research”. The number of publications has increased rapidly during the past 25 years. Based on the number of publications, the algorithm predicted that “Immunotherapy”, Prognostic research”, “Protein expression”, “Case reports”, “Gemcitabine and mechanism”, “Clinical study of gemcitabine”, “Operation and postoperation”, “Chemotherapy and resection”, and “Review and management” as current research topics. To our knowledge, this is the first study on this subject of pancreatic cancer research, which has become possible due to the improvement of algorithms and hardware.

## Introduction

Pancreatic ductal adenocarcinoma, referred to as pancreatic cancer, is one of the most malignant tumors due to its late diagnosis and early metastasis (1). It is currently ranked 11^th^ among the most common cancers globally (2). Despite long-term efforts worldwide, the treatment of patients with pancreatic cancer remains unsatisfactory. The only cure is a complete tumor resection, but only 15–20% of patients are candidates for tumor resection (3). In most cases, the diagnosis is not made until the disease is at an advanced, metastatic stage, and thus, the vast majority of patients can only be treated by palliative strategies (3). Thus, the survival time of patients with stage 1 disease is better than that of patients diagnosed at more advanced metastatic stages. Whereas the overall 5-year survival rate of all stages is proposed to be as low as 3–15% (2, 4), the survival times are up to 54 months for resectable, up to 15 months for localized, and up to 12 months for advanced, metastatic pancreatic cancer (5–8). Although survival is still poor, progress has been made by high-quality, multicenter randomized controlled trials initiated, e.g., by the European Study Group of Pancreatic Cancer (ESPAC), which demonstrated that the combination of gemcitabine with capecitabine significantly prolonged median overall survival to 28.0 months after resection compared to 25.5 months in the gemcitabine group alone (9, 10). More recently, data from the French-Canadian Uni-Cancer GI PRODIGE 24/CCTGPA.6 trial demonstrated that a modified FOLFIRINOX chemotherapy regimen improved the survival time to 54.4 months in patients with macroscopically resected pancreatic cancer compared to 35 months in the gemcitabine monotherapy group (5, 7). To further improve this still devastating prognosis, novel treatment strategies focus on developing more effective medication targeting metastasis, hypoxia, and immunosuppression.

Research results are usually reported in publications, but they have increased so much that it is hardly manageable. Before January 1996, only 21,430 publications dealing with pancreatic cancer were available, and the number increased to 60,296 in October 2021. Researchers are unable to read this massive amount of literature. New tools are needed to extract and summarize the most relevant information from the vast literature to reflect the current research interests and trends in public attention (11). The so-called “bibliometrics” is a quantitative analysis method of academic publications. It can discover the progress of disciplinary research from a macro perspective and provide support for future research directions (12). A previous bibliometric study in 2020 analyzed 1,171 publications to extract information on the use of nanoparticles in pancreatic cancer research (13). The available data revealed that although most research on nanoparticles in pancreatic cancer is isolated, there is little attempt to combine the findings with other disciplines. Due to the lack of tools to analyze massive amounts of documents in the past, the analysis of large-scale publications on pancreatic cancer has not been described at present.

Recent algorithms and hardware improvements can now be processed by larger datasets (14). In particular, the availability of the computing technology “Natural Language Processing” (NLP) in machine learning is extremely useful for the detailed analysis of medical information (15, 16). Additionally, latent Dirichlet allocation (LDA), one of the most classical topic modeling methods in bibliometrics, facilitates the screening of large datasets of unstructured text (12). LDA creates a term function vocabulary based on the frequency of words coexisting in the document set. After creating vocabulary words, LDA determines the probability that the article belongs to a specific topic according to the frequency of vocabulary appearing in each document. We have previously constructed a set of LDA and NLP methods to analyze more than 23,000 publications from 1994 to 2018 related to rectal cancer and successfully predicted the research deficiencies in the last 25 years. Likewise, we predicted potential future research focuses, namely, “Basic Research”, “Inflammation”, and others. Our analysis also confirmed that rectal cancer should be treated independently from colon cancer (17). To our knowledge, a similar study on pancreatic cancer has not yet been published.

The present study analyzed changes in pancreatic cancer research topics from January 1996 to October 2021 *via* machine learning and NLP techniques. Based on our previous data with rectal cancer, we improved the algorithm to analyze the publications in more detail, express them more visually, find hard evidence in each research area, and identify potential future research trends in pancreatic cancer research.

## Results

### The Number of Publications in Pancreatic Cancer Research Increases Every Year

**Figure 1:**
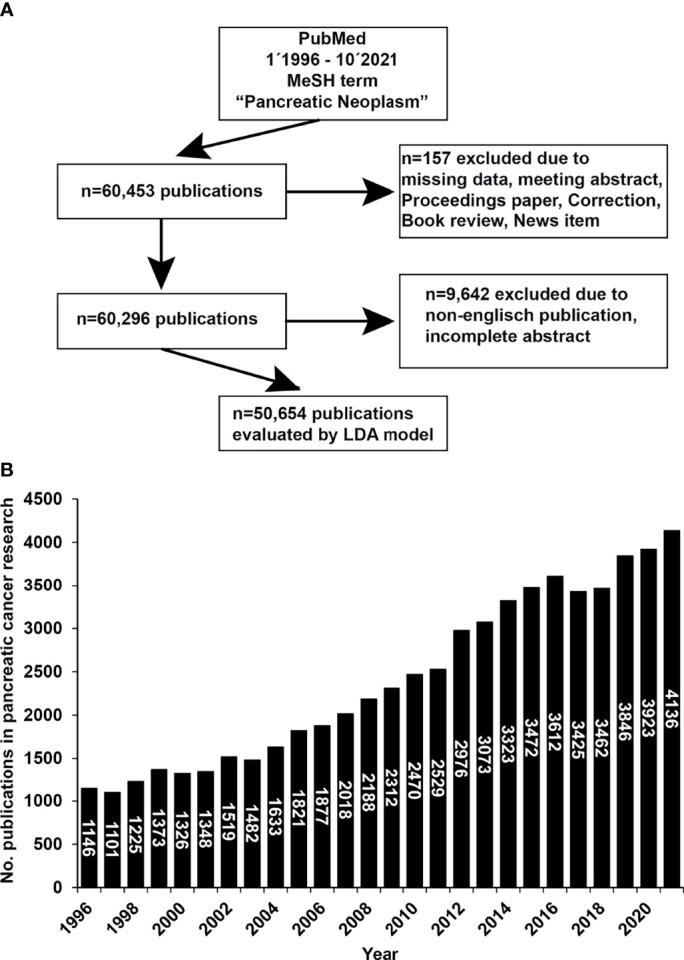
The number of publications on pancreatic cancer has increased rapidly in the past 25 years. **(A)** PubMed publications from January 1^st,^ 1996 to October 10^th,^ 2021 were screened and downloaded using the R package easyPubMed and the MeSH term “Pancreatic Neoplasms”. From the initially identified 60.453 publications, 157 publications were excluded manually due to missing data or when the publication was a meeting abstract, proceedings paper, a correction, a book review, or a news item. Of the resulting 60,296 publications, another 9,642 were manually excluded when the language was not English or the abstract was incomplete, resulting in 50,654 publications. **(B)** The selected 50,654 publications were analyzed by LDA and Python. The data were visualized with Excel and R. The number of publications (No. publications) per year is shown.

From 1996 to 2021, 60,453 publications were identified under the Medical Subject Headings (MeSH) term “Pancreatic Neoplasms”, as shown by a flowchart (**Figure 1A**). We manually excluded 157 publications from further analysis, and the exclusion criteria were missing data if the publication was a meeting abstract, a proceedings paper, a correction, a book review, or a news item. The number of the residual 60,296 publications was further diminished by the manual exclusion of non-English publications or publications with incomplete abstracts, resulting in 50,654 remaining publications. The LDA bibliometric modeling method further analyzed these selected publications as explained in detail (**Supplemental Information 1**). We found that 1,146 publications in pancreatic cancer research were available in 1996. This number increased to 3,923 publications in 2020 and 4,136 in 2021, as shown by a diagram (**Figure 1B**). In the last 25 years, an average of 2,411 publications were published each year, while the average growth rate was 5.42%.

To further subdivide research focuses in pancreatic cancer research, we identified eight categories: “Systematic Review,” “Meta-Analysis”, “Multicenter Study”, “Letter”, “Comment”, “Clinical Trial”, “Review”, and “Case Reports”, along with the number of publications according to the database-provided areas in pancreatic cancer research (**Table S1**). We found, for example, 552 publications in the field of Clinical Trials in the period 1996 to 2000 and 643 publications in the period 2016 to 2021. As shown in a diagram, the category “Clinical Trial” contained an average of 90 publications per year, and the rate increased by 1.7 publications per year (**Figures 1**, **S1**). We further analyzed the relationship between the publication of clinical trials and countries. The United States ranked first with 553 publications on clinical trials, accounting for 27% of the total publications, followed by Japan with 316 publications, accounting for 15%, followed by Italy with 178 publications, accounting for 9%, and Germany with 148 publications, accounting for 7% (**Table S2**). Likewise, the number of publications in the “Multicenter Study” section was 82 from 1996 to 2000 but increased to 656 from 2016 to 2021 (**Table S1**), indicating exponential growth. Although the number of published “Multicenter Studies” was initially lower than that of “Clinical Trials”, it surpassed the publication number of Clinical Trials in 2018 (**Figure S1B**). Additionally, we found that in 2020, 37 of the 118 Clinical Trials published were multicenter studies, accounting for 31.4% (**Table S3**). In contrast, in 2010, 90 manuscripts were published as Clinical Trials, and only 21 were also multicenter studies, namely, 23.3%. This phenomenon suggests that many clinical trials are currently performed as multicenter studies. Interestingly, research in “nutrition and cachexia” seems to be a relatively new field of research, as the number of publications increased from approximately 2 publications per year in the period 1996 to 2015 to 19 publications in 2016 and reached 131 publications in 2019 (**Figure S2A**). Additionally, the number of publications dealing with immunotherapy in pancreatic cancer strongly increased and reached a level of 329 publications in 2019 (**Figure S2B**), suggesting that immunotherapeutic approaches are currently being intensively researched.

### The US, China, and Japan Have the Highest Number of Publications in Pancreatic Cancer Research

**Figure 2:**
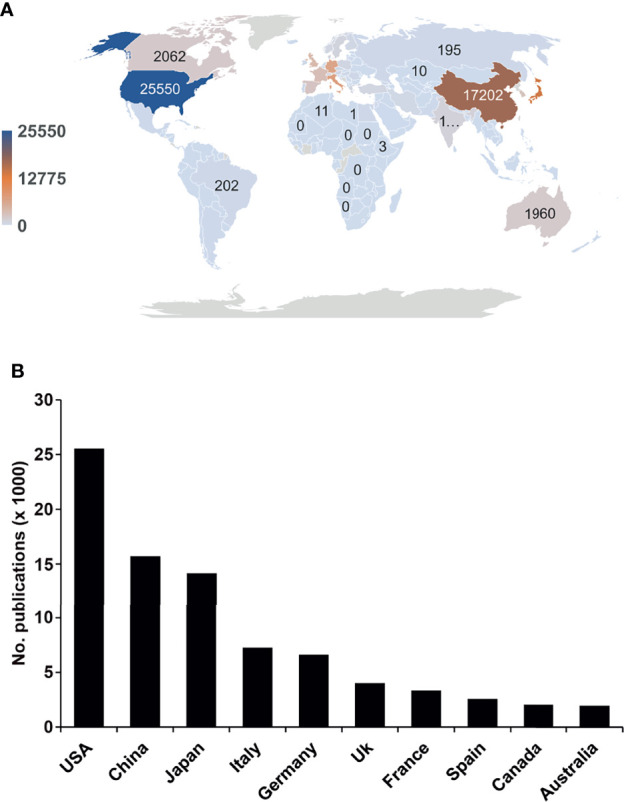
Global pancreatic cancer research differs significantly between regions. **(A)** The global distribution of pancreatic cancer publications in the last 25 years by number is shown. We extracted country information based on the publication’s affiliation. The darker the color is, the greater the volume of publications. Significant head effect: The number of posts in the Northern Hemisphere is much higher than that in the Southern Hemisphere. **(B)** Top 10 countries with the highest publication numbers in pancreatic cancer research.

Next, we analyzed the authors’ affiliation information to evaluate the geographical distribution of the publications available in pancreatic cancer research. We found that 113 countries or regions had published manuscripts (**Figure 2A**). Of all publications, the United States, China, Japan, Italy, and Germany accounted for 24.9%, 15.3%, 13.9%, 7.1%, and 6.5%, respectively. The top 10 countries with the highest number of publications account for 81.1% of all publications (**Figure 2B**). Africa and Southeast Asia contributed less than 5% to the total number of publications. According to geographic data, the vast majority of the world’s population has not yet participated in pancreatic cancer research.

### The MeSH Terms “Pathology” and “Metabolism” Occur Most Frequently

**Figure 3:**
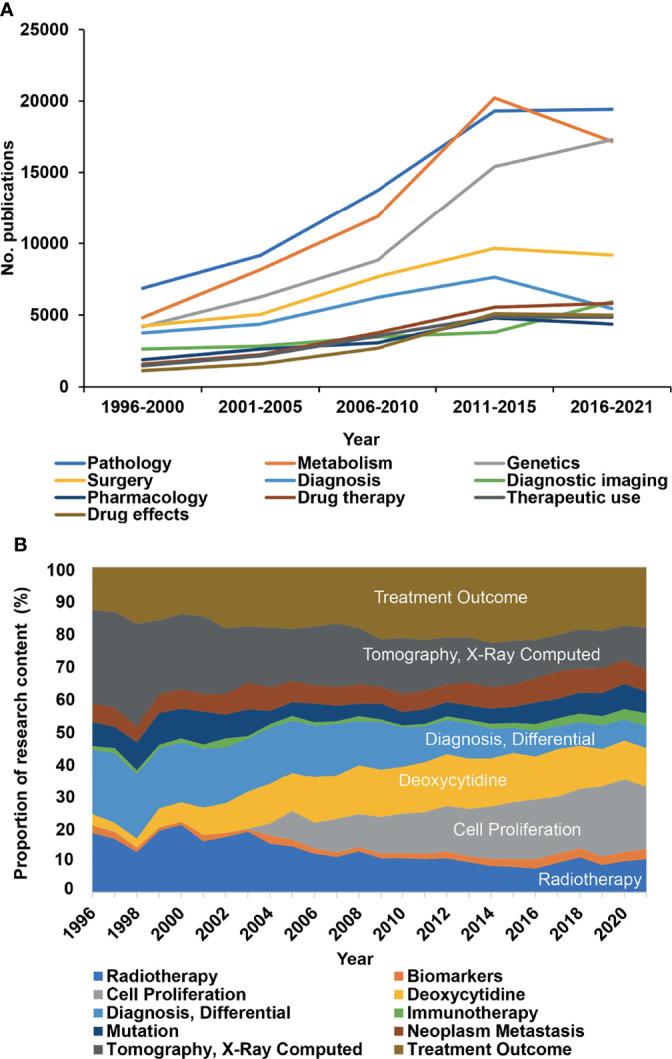
The MeSH terms “Pathology” and “Metabolism” occur most frequently. R extracted significant MeSH terms from publications listed in the PubMed database according to the publication topic. **(A)** Ten of the most widely studied MeSH terms and their number (No.) of publications per year. “Pathology”, “metabolism”, and “surgery” were the most frequently studied research fields in the last 25 years. **(B)** Proportional changes in some representative MeSH terms are given in percent (%) per year. The MeSH terms are marked by different colors as indicated.

**Figure 4:**
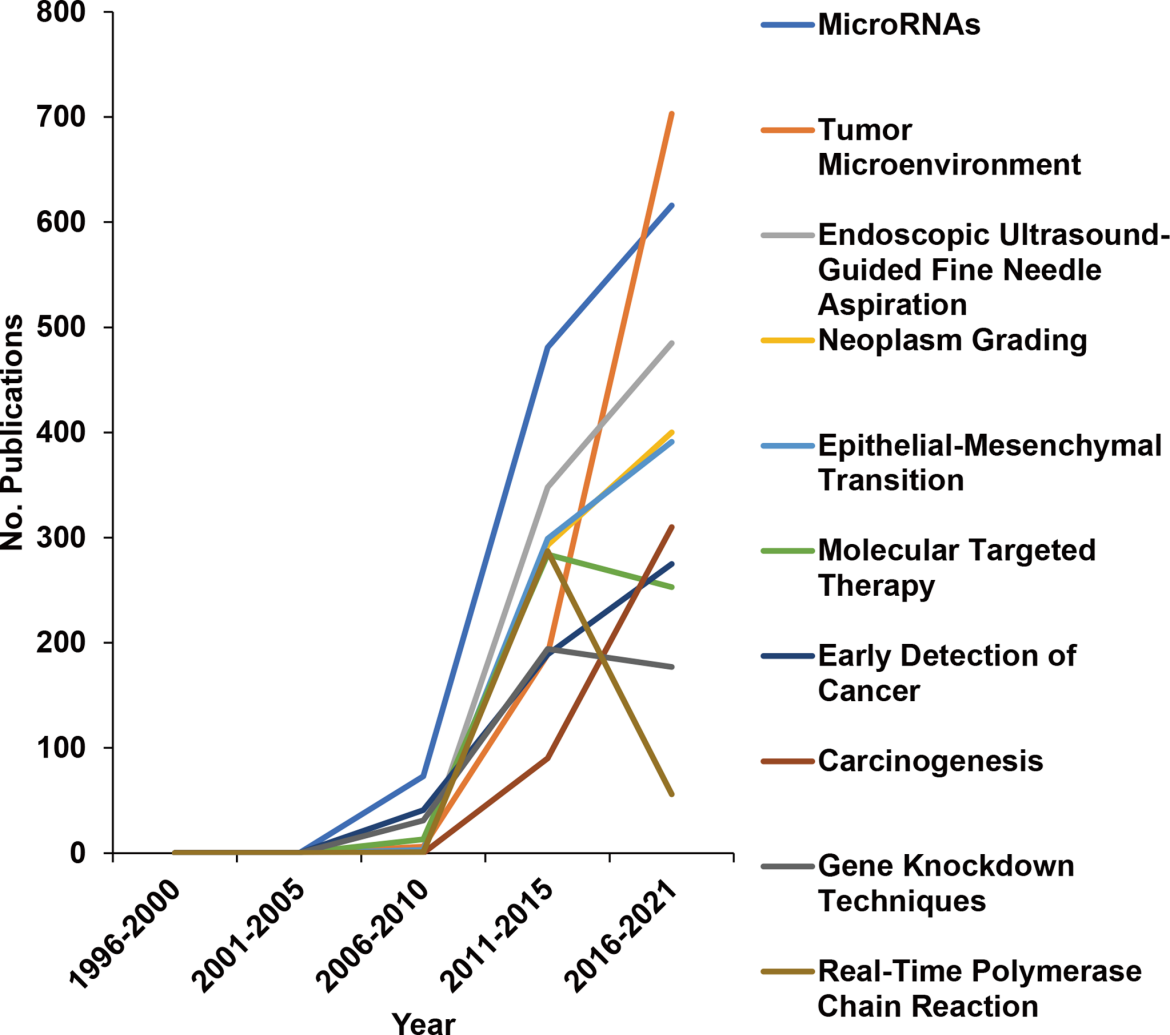
Changes in the 10 most widely studied newly merged MeSH terms over the years. MeSH terms were extracted by R, and newly merged MeSH terms that appeared after 1996 are shown as the number (No.) of publications per year.

To further explore changes in research directions, we analyzed 12,059 MeSH terms related to pancreatic cancer research in a total of 1,309,154 repetitions from 1996 to 2021. Based on the highest frequency, we selected the terms “Pathology”, “Metabolism”, “Genetics”, and “Surgery” (**Figure 3A**, new **Table S4**), indicating that these directions are studied most intensively. To further discover research on genes related to “Metabolism” and to identify the number of related manuscripts, we conducted a search and found that BRAC2, BRAC1, KRAS, and other genes were the most common, widely studied genes (new **Table S5**). Then, representative MeSH terms were selected and further analyzed by studying their proportions. Whereas the number of publications in the research fields “Radiotherapy”, “Tomography, X-ray Computed”, and “Diagnosis, Differential” constantly decreased from 1996 to 2021; “Cell Proliferation”, “Deoxycytidine”, and “Treatment Outcome” emerged (**Figure 3B**), suggesting that these changes represent current research focuses. Additionally, the proportion of publications in the field of “Immunotherapy” emerged, although the number of publications remains limited. We also analyzed the newly emerging research directions as MeSH terms that appeared after 1996, which accounted for 42.5% of the total number of MeSH terms but appeared only 6,933 times (**Figure 4**). Among these MeSH terms, the number of publications with the topics “MicroRNAs” and “Tumor Microenvironment” has grown tremendously, as concluded to the appearance of these topics in 2006 and 2010, with only 2 and 8 publications, respectively. Nevertheless, by 2020, the number of publications with “MicroRNAs” and “Tumor Microenvironment” reached 616 and 703, respectively. The current research focuses on many new technologies represented by these emerging MeSH terms, which have appeared frequently since 1996, indicating that their research has been conducted more in-depth.

### LDA Identified the Main Areas “Treatment Study”, “Clinical Manifestations and Diagnosis”, “Review and Management”, and “Basic Research”

**Figure 5:**
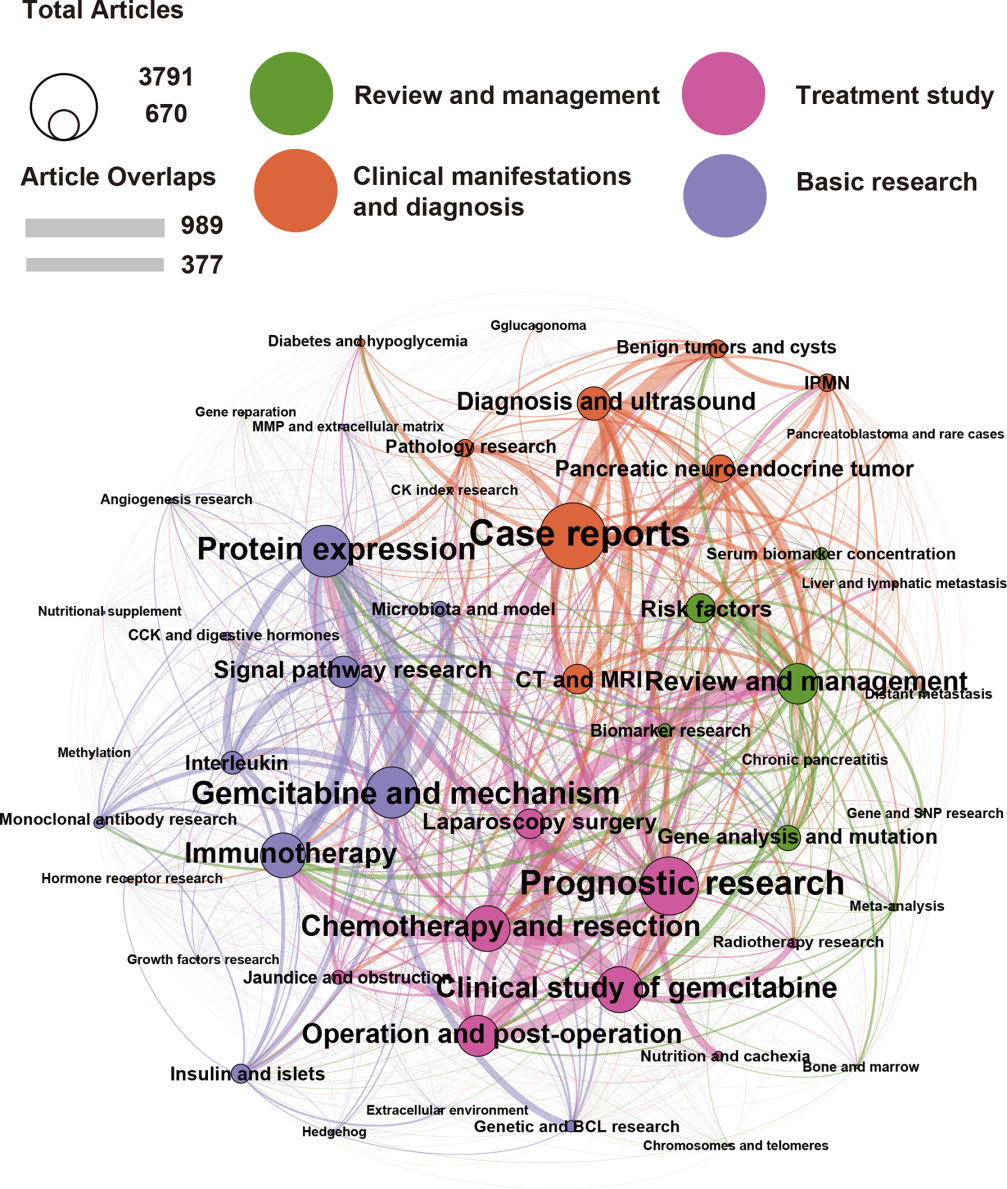
The main areas in pancreatic cancer research over the last 25 years are “Treatment Studies”, “Clinical Manifestations and Diagnosis”, “Review and Management”, and “Basic Research”. We analyzed pancreatic cancer publications from the last 25 years using the LDA algorithm, from which 50 topics were identified and classified into four broad categories: “Clinical Manifestations and Diagnosis” (orange), “Review and Management” (green), “Treatment Study” (pink), and “Basic Research” (purple). The circle size represents the number of publications contained in the research topic. For example, the algorithm found that the topic case reports contain 3,791 publications. The line thickness between the circles represents the degree of overlap between the two research topics, such as the 989 publications with the highest degree of overlap in “Operation and postoperation” and “Laparoscopy surgery”.

Given the wide range of MeSH terms in publications, it was not easy to express them intuitively. To solve this problem, we used the LDA algorithm to generate relevant topics based on the content of each publication. We expect to reflect on the research topics over the past 25 years in this approach. To express the interrelated topic clusters and the relationship between essential topics, we used topic network analysis and visualization analysis by LDA and the Louvain algorithm, as previously described (18). We found that the last 25 years of pancreatic cancer research can be summed up in the following four main topics that had the highest number of publications: “Treatment Study”, “Clinical Manifestations and Diagnosis”, “Review and Management”, and “Basic Research” (**Figure 5**). In addition, each topic covered many small branches, as evident from the colors of each main topic indicated by large circles and related smaller circles of the same color. For example, the main, orange-marked cluster “Clinical Manifestations and Diagnosis of Pancreatic Cancer” can be subdivided into the branches “Case Reports”, “Computed Tomography (CT) and Magnetic Resonance Imaging (MRI)”, “Diagnosis and Ultrasound”, “Pancreatic neuroendocrine tumor”, “Benign tumors and cysts”, “IPMN”, “Pathology Research”, and other areas, whose circle sizes are determined by the number of publications. It should be mentioned that there were strong relationships between each topic, as indicated by connecting lines. Therefore, “Case Reports” have, e.g., strong connections with “Prognostic research”, “Review and Management”, “Pathology Research”, and so on, and the thickness of the connecting lines determines the number of interactions. The next main and green-labeled cluster, “Review and Management” covers the main branch “Review and Management” and the smaller branches “Risk Factors”, “Gene Analysis and Mutation”, “Serum Biomarker concentration”, “Biomarker research”, “Chronic pancreatitis”, and “Gene and SNP research”. This cluster may be interpreted to play a major role in combining basic and clinical research, as there are strong connecting lines to the subbranches of other main clusters, such as “Immunotherapy”, “Clinical study of gemcitabine”, “Protein expression”, and “Signal pathway research”. The pink labeled “Treatment Study” cluster with its main branch “Prognostic research” and subbranches “Chemotherapy and resection”, “Clinical study of gemcitabine”, “Operation and postoperation”, “Laparoscopy surgery”, “Jaundice and obstruction”, “Nutrition and cachexia”, and others, are strongly related to branches of all other main clusters, as indicated by strong connection lines. The relatively small violet bubbles for “nutrition and cachexia” and “jaundice and obstruction” may indicate that there is little research on hospice care, and to some extent, there is a lack of research on the perspective of patients and patients’ psychological construction. The violet-labeled “Basic Research” cluster and its main branches “Protein expression”, “Gemcitabine and mechanism”, and “Immunotherapy” have many subbranches, such as “Insulin and Islets”, “Interleukin”, “Microbiota and model”, “Angiogenesis research”, “MMP and extracellular matrix”, “CCK and digestive hormones”, “Monoclonal antibody research”, “Growth factor research”, “Hedgehog”, Extracellular environment”, and “Genetic and BCL research”. This cluster “Basic Research” has very small or missing connecting lines to the more clinically orientated branches, suggesting that patient or clinically orientated research is the exception and could be improved in the future. In summary, research on pancreatic cancer has covered a wide range of clinical, therapeutic, and basic research.

### LDA Results: “Immunotherapy” and “Prognostics” Are Research Focuses

**Figure 6:**
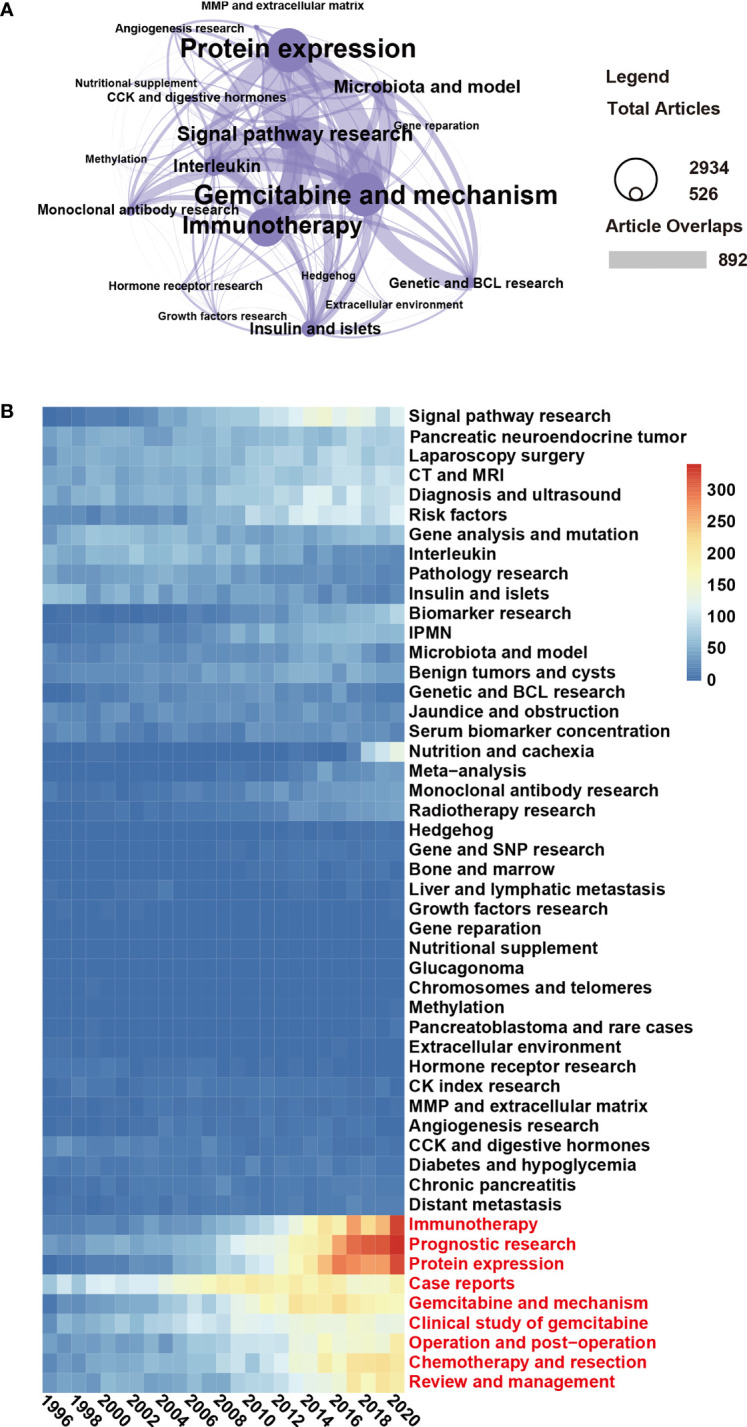
“Immunotherapy” and “Prognostic research” are current research topics. **(A)** Topic cluster network of the topic “Basic research” and the interrelation of subtopics was analyzed using the LDA algorithm. In this cluster, the “gemcitabine and mechanism” topic included 2,934 publications, and 892 of these publications shared the same topic with the topic “protein expression”. **(B)** The heatmap presents the number of publications per year of 50 research topics over the past 25 years. The data were generated by the use of the LDA algorithm. The abscissa represents the year, the ordinate represents the topics, and the color brightness represents the number of publications and reflects the shift in research focus.

According to the network cluster based on the number of publications in the last 25 years, the current trends in basic pancreatic cancer research are “Protein expression”, Gemcitabine and mechanism”, “Immunotherapy”, “Signal pathway research”, “Microbiota and model”, “Interleukin”, “Insulin and islets” and many smaller research fields (**Figure 6A**, compare **Figure 5**). To reflect the general research topics in pancreatic cancer, we visualized the LDA results by a heatmap that shows 50 important research topics of the last 25 years and the publications per year. Whereas the number of publications in the fields “Pathology research” and “Insulin and islets” decreased, “Immunotherapy”, “Prognostic research”, “Protein expression”, “Case reports”, “Gemcitabine and mechanism”, “Clinical study of gemcitabine”, “Operation and postoperation”, “Chemotherapy and resection”, and “Review and management” increased dramatically in recent years (**Figure 6B**). Therefore, the latter topics may be current, and future research focuses. However, the decreasing number of publications in “Pathology” and “Insulin and islets” suggests that these research directions may have changed.

## Discussion

We used machine learning and NLP to analyze 60,296 PubMed articles published from 1996 to 2021 in the field of pancreatic cancer. Additionally, we visually displayed and analyzed the results from a macroscopic perspective. To the best of our knowledge, this is the only analysis of its kind to be reported. We found that in the past 25 years, the number of pancreatic cancer-related scientific publications increased from 21,430 in 1996 to 82,292 in 2021, and the research content has become more extensive. In the last 25 years, pancreatic cancer research has mainly focused on the MeSH terms “Clinical Manifestations and Diagnosis”, “Review and Management”, “Treatment Study”, and “Basic Research”. The research topics have changed over the years, and the current research focuses are predicted to be “Immunotherapy” and “Prognosis research”.

Currently, bibliometric analysis can only be performed with a few software tools, such as VOSviewer, Bibliographic Items Co-occurrence Matrix Builder (BICOMB), and CiteSpace (19–21). Unfortunately, the analysis of research topics is mostly based on keywords marked in the publications rather than on their content. In addition, this software has problems analyzing large amounts of text due to memory and format limitations. In contrast, the method we developed is based on LDA, an unsupervised learning algorithm. There is no need to label the training set; our method can easily analyze a large amount of text. In addition, we improved the visualization of the results, making the detected trends and changes more intuitive to analyze.

We found that “Prognosis research” research is a study focus. Nevertheless, the 5-year survival rate of pancreatic cancer has increased by only 3–5% (22). In contrast, and due to breakthroughs in early precancerous diagnosis, the 5-year survival rate of breast and colorectal cancer has been greatly improved (17, 23). The reason may be that early diagnosis of pancreatic cancer is challenging due to its unique biological characteristics, including the tumor’s location deep within the abdominal cavity, the lack of ideal biomarkers, and an almost late diagnosis in the advanced, metastatic stage (1). Based on publications dealing with the early diagnosis of pancreatic cancer and published over the past 25 years, only 1–2% describe innovative diagnosis techniques, such as “Detection of Circulating Tumor DNA”, “Artificial Intelligence”, “Wearable Cancer Monitors”, and other new technologies (24, 25). The MeSH term “Clinical Manifestations and Diagnosis” of pancreatic cancer matches to more than 12,000 publications, of which case reports have the most significant number of publications. However, case reports remain the research method with the lowest clinical reliability and validity. Our data demonstrate that this term has a loose connection to other topics in pancreatic cancer research, suggesting a considerable gap between mechanistic research and clinical diagnosis and practice. Nevertheless, with more feasible technologies and new algorithms, the classification of pancreatic cancer into different subtypes, as described, may enable new tumor diagnosis and treatment (26).

The MeSH term “Basic Research” has been another hot area in the last 25 years of pancreatic cancer research. Many studies have focused on “immunotherapy” and its underlying mechanisms (27). There are few reports of the combination of immunotherapy with chemotherapy and chemoradiation, which has improved the overall survival of patients with pancreatic cancer (28–30). Unfortunately, most of the reported phase 1 and 2 clinical trials in immunotherapy failed or did not achieve the expected results in pancreatic cancer treatment (31). We thus put forward our views on the ineffectiveness of immunotherapy in pancreatic cancer. First, too few studies on pancreatic cancer’s immune and inflammatory mechanisms. People have observed spontaneous melanoma regression, found that the cause is closely related to the patient’s immune and inflammatory response, and successfully used immunotherapy in melanoma. We found only 297 publications related to the MeSH term “inflammation” in pancreatic cancer. Although pancreatitis is associated with a vigorous immune response and even systemic inflammation, there is an “immune desert” phenomenon in pancreatic cancer, which means immune suppression caused by an extremely dense extracellular matrix and, therefore, the failure of immune cells to infiltrate the tumor (32). Therefore, pancreatic cancer can develop micrometastases very early, resulting in rapid disease progression. Accordingly, increasing the number of infiltrating T cells and relieving immunosuppression in pancreatic cancer is a current and future focal point of research interest.

Our analysis identified that the microbiome in pancreatic cancer is gradually becoming a research focus, with more than 872 publications discussing related issues. At present, publications report that the composition of the oral, gut, or tumor microbiome is closely related to immune quality and infiltration (33–37). Therefore, in the future, a more intense examination of the microbiome’s function may require early diagnosis and immunotherapy of pancreatic cancer. The microbiome is an integral part of the pancreatic cancer environment and plays a pivotal role in cancer development and progression. Microbe-induced inflammation affects oncogenic signaling, tumor cell metabolism, and immune responses in pancreatic cancer. Microbiome analysis can serve as a biomarker and identify individuals at risk for pancreatic cancer. Microbiome modulation combined with immunotherapy is a novel approach, although further studies are required to confirm its usefulness in pancreatic cancer in the future. On the other hand, microbiome research is still in its infancy, and the number of publications in this field is limited, thus counteracting the analysis of massive amounts of data.

Although the extracellular matrix plays a significant role in carcinogenesis and treatment resistance of pancreatic cancer (38), less than 4% of publications related to the topic show that the research is still in the stage of scarcity. Recently, several new chemotherapy drugs targeting the extracellular matrix, including PEGPH2 (39), an enzyme targeting matrix hyaluronic acid, pegilodecakin (40), a PEGylated IL-10, ibrutinib, a Bruton tyrosine kinase inhibitor, and napabucasin (41), a STAT3 inhibitor targeting cancer stem cells, were explored in clinical trials. Unfortunately, the results did not meet the expectations. More dimensional and complex research may be required to explore the exceptional multicellular environment of the pancreas.

Finally, we would like to emphasize the limitations of our study. First, all included publications are from the PubMed database, which is inherently biased. Second, this manuscript cannot show the entire amount of acquired data due to its high complexity and space limitations. We only provided an analytical approach from the NLP perspective and superficial insights into pancreatic cancer research topics. However, we believe that machine learning and NLP are new tools for scientists to extract objective and comprehensive clues from large amounts of data.

## Conclusion

The number of publications in pancreatic cancer research has increased rapidly during the past 25 years, and it is a great challenge for scientists to filter out important information from these exploding publication numbers. Using machine learning and NLP, we identified the research topics that developed from “Interleukin and Pathology” in the past to “Immunotherapy”, “Prognostics”, “Microbiome”, “Early Diagnosis”, and “Molecular Typing” in the present and future. We conclude that future breakthroughs in pancreatic cancer research may depend on understanding complex relationships, new technologies, and the application and popularization of new and innovative diagnostic technologies. We found only a few studies with the topics “Hospice Care”, “Quality of Life”, “Patient Perspectives”, and “Economics”, suggesting that these fields are not currently being intensively researched. Machine learning and NLP may be a handy new tool for scientists to extract objective and comprehensive clues from large amounts of data.

## Materials and Methods

### Screening of Publications and Access

The pubquery package in R (https://www.r-project.org/, version: 4.1.1) was used to access and download all the metadata. All PubMed publications from January 1st, 1996 to October 1st, 2021 were searched with the MeSH term “Pancreatic Neoplasms”. A MeSH term is a controlled vocabulary thesaurus produced by the National Library of Medicine (https://www.ncbi.nlm.nih.gov/mesh). It consists of terms naming descriptors in a hierarchical structure that permits searching various specificity levels and enables search publications to obtain as complete data as possible. A complete record of the search results is downloaded in XML format. Visualizations are mostly based on Excel (Microsoft Corporation, Redmond, WA, USA). The search and download code is available in R by the easyPubMed package (https://cran.r-project.org/web/packages/easyPubMed/index.html).

### Algorithms and Analysis Methods

Python was designed in the early 1990s by Guido van Rossum of the Dutch Society of Mathematics and Computer Science Research as an alternative to the ABC language (https://docs.python.org/zh-cn/3/license.html). Python provides efficient high-level data structures and simple and effective object-oriented programming and is widely used in NLP and other analyses. Python (https://www.python.org/, version 3.7.1) was used to extract detailed publication data, including publication year, region, abstract, research type, and MeSH terms of each article. LDA was used to identify more specific research topics and model them by analyzing the abstract of all indexed articles in the record and setting the number of identified topics to 50. The criteria of topic number selection are based on proper perplexity, redundancy, and legibility. Based on the topic probability calculated by the algorithm, we manually determined the topic of each article. Heatmaps were used to describe the research topics and publication dates. We used the Louvain algorithm in Gephi software (https://gephi.org/, version 0.9.2) for clustering analysis to establish the topic network to determine the relationship between topics. We identified two topics with the highest attribution probability in each article and calculated the number of these two topics appearing simultaneously in each document. Finally, we established links between all the topics. LDA-related code can be found in the **Supplemental Information**.

## Data Availability Statement

The original contributions presented in the study are included in the article/**Supplementary Material**. Further inquiries can be directed to the corresponding author.

## Author Contributions

Conceptualization: KW and IH. Methodology: KW. Formal analysis: KW. Writing: KW and IH. All authors contributed to the article and approved the submitted version.

## Funding

This study was supported by a scholarship to KW from the China Scholarship Council (202006370023). IH was supported by grants from the German Research Council (DFG HE 3186/15–1), Karsten Burmeister—BIMAG Bau- und Industriemaschinen GmbH, Heidelberger Stiftung Chirurgie, Dietmar Hopp-Stiftung, Klaus Tschira Stiftung and Hanns A. Pielenz-Stiftung.

## Conflict of Interest

The authors declare that the research was conducted in the absence of any commercial or financial relationships that could be construed as a potential conflict of interest.

## Publisher’s Note

All claims expressed in this article are solely those of the authors and do not necessarily represent those of their affiliated organizations, or those of the publisher, the editors and the reviewers. Any product that may be evaluated in this article, or claim that may be made by its manufacturer, is not guaranteed or endorsed by the publisher.

## Abbreviations

CT, Computed tomography; LDA, Latent Dirichlet Allocation; MRI, Magnetic resonance imaging; MeSH, Medical subject headings; NLP, Natural language processing.
