# Supplementary material for: Machine-Learning-Based Bibliometric Analysis of Pancreatic Cancer Research Over the Past 25 Years
Source: Front Oncol. 2022 Mar 28;12:832385. doi: 10.3389/fonc.2022.832385 (PMC8995465; doi:10.3389/fonc.2022.832385)
Supplement: Supplementary file 1 [file DataSheet_1.pdf]

Supplement

Machine-learning based bibliometric analysis of pancreatic cancer research over the past 25 years

Kangtao Wang & Ingrid Herr

Table of Content

Table S1 Changes in the number of publication types from 1996 to 2021

Table S2 Number, country, percentage and ranking of publications in the field of "Clinical trials" from 1996 to 2021

Table S3 Number of publications in the fields "Multicenter Study" and "Clinical Trial" from 1996 to 2021 and changes in their proportions

Table S4 Most widely studied top 20 MeSH terms in pancreatic cancer research

Table S5 The 10 most studied genes in PDAC Metabolism studies and their basic information

Fig. S1 The number of published Clinical Trials and Multicenter Studies increased

Fig. S2 The number of publications in the fields "Nutritiona and cachexia" and "Immunotherapy" increased

Supplemental information 1 Core code of LDA methods

Table S1 Changes in the number of publication types from 1996 to 2021

Publication Type	Number of publications/years				
	**1996-2000**	**2001-2005**	**2006-2010**	**2006-2010**	**2016-2021**
**Case Reports**	1,323	1,584	2,176	2,248	2,149
**Review**	1,138	1,365	1,659	2,222	2,611
**Clinical Trial**	522	630	435	610	643
**Comment**	149	186	365	665	951
**Letter**	224	228	522	645	691
**Multicenter Study**	82	131	222	434	656
**Meta-Analysis**	5	17	68	279	479
**Systematic Review**	2	11	61	163	457

Table S2 Number, country, percentage and ranking of publications in the field of "Clinical trials" from 1996 to 2021

Country	No. Clinical trails	Percentage	Rank
**USA**	553	27	1
**Japan**	316	15	2
**Italy**	178	9	3
**Germany**	148	7	4
**UK**	120	6	5
**China**	143	7	6
**France**	85	4	7
**Netherlands**	82	4	8
**Korea**	65	3	9
**Spain**	38	2	10
**Australia**	36	2	11
**Canada**	35	2	12
**Switzerland**	33	2	13
**Greece**	33	2	14
**Sweden**	25	1	15

Table S3 The number of publications in the fields "Multicenter Study" and "Clinical Trial" from 1996 to 2021 and changes in their proportions

Year	Multicenter Study	Clinical Trial	Both	Percentage
**1996**	20	59	14	24
**1997**	12	55	8	15
**1998**	12	66	5	8
**1999**	18	81	12	15
**2000**	20	83	12	14
**2001**	18	72	14	19
**2002**	22	75	17	23
**2003**	24	77	19	25
**2004**	29	92	20	22
**2005**	38	93	18	19
**2006**	33	77	15	19
**2007**	41	78	15	19
**2008**	60	89	25	28
**2009**	38	75	17	23
**2010**	50	90	21	23
**2011**	74	102	28	27
**2012**	87	130	36	28
**2013**	93	105	38	36
**2014**	81	110	30	27
**2015**	99	119	36	30
**2016**	106	114	35	31
**2017**	98	105	33	31
**2018**	110	103	27	26
**2019**	160	108	39	36
**2020**	132	118	37	31
**2021**	50	50	14	28

Table S4 Most widely studied top 20 MeSH terms in pancreatic cancer research

Mesh terms	Number of indicated MeSH terms/years				
	1996-2000	2001-2005	2006-2010	2011-2015	2016-2021
**Pathology**	6,873	9,163	13,739	19,300	19,420
**Metabolism**	4,832	8,164	11,885	20,219	17,176
**Pancreatic Neoplasms**	5,927	7,528	10,486	14,963	15,908
**Humans**	5,681	7,324	10,333	14,778	15,946
**Genetics**	4,187	6,257	8,828	15,417	17,302
**Surgery**	4,243	5,044	7,675	9,648	9,189
**Diagnosis**	3,764	4,375	6,237	7,637	5,455
**Female**	2,924	3,632	5,278	7,771	7,797
**Male**	2,917	3,595	5,119	7,534	7,660
**Middle Aged**	2,421	2,953	4,318	5,987	6,164
**Diagnostic Imaging**	2,641	2,845	3,479	3,808	5,931
**Pharmacology**	1,895	2,635	3,079	4,805	4,380
**Aged**	2,090	2,615	3,746	5,371	5,785
**Methods**	1,263	2,331	3,890	6,070	6,944
**Drug Therapy**	1,588	2,246	3,780	5,544	5,818
**Therapeutic Use**	1,481	2,158	3,560	4,907	4,850
**Adult**	1,727	2,124	2,821	3,,750	3,483
**Complications**	1,410	1,848	2,318	2838	2,180
**Administration & Dosage**	1,130	1,756	2,609	3,893	3,385
**Therapy**	1,299	1,751	2,556	3,732	3,944

Table S5 The 10 most widely studied genes in PDAC Metabolism studies and their basic information

Gene Symbol	More information	Relevance score	related publications
**BRCA2**	BRCA2 DNA Repair Associated	9	405
**BRCA1**	BRCA1 DNA Repair Associated	9	278
**TP53**	Tumor Protein P53	8	518
**ATM**	ATM Serine/Threonine Kinase	6	144
**PALB2**	Partner And Localizer Of BRCA2	6	116
**CHEK2**	Checkpoint Kinase 2	5	37
**KRAS**	KRAS Proto-Oncogene, GTPase	5	2628
**CDKN2A**	Cyclin Dependent Kinase Inhibitor 2A	5	584
**CDH1**	Cadherin 1	5	104
**PTEN**	Phosphatase And Tensin Homolog	5	184

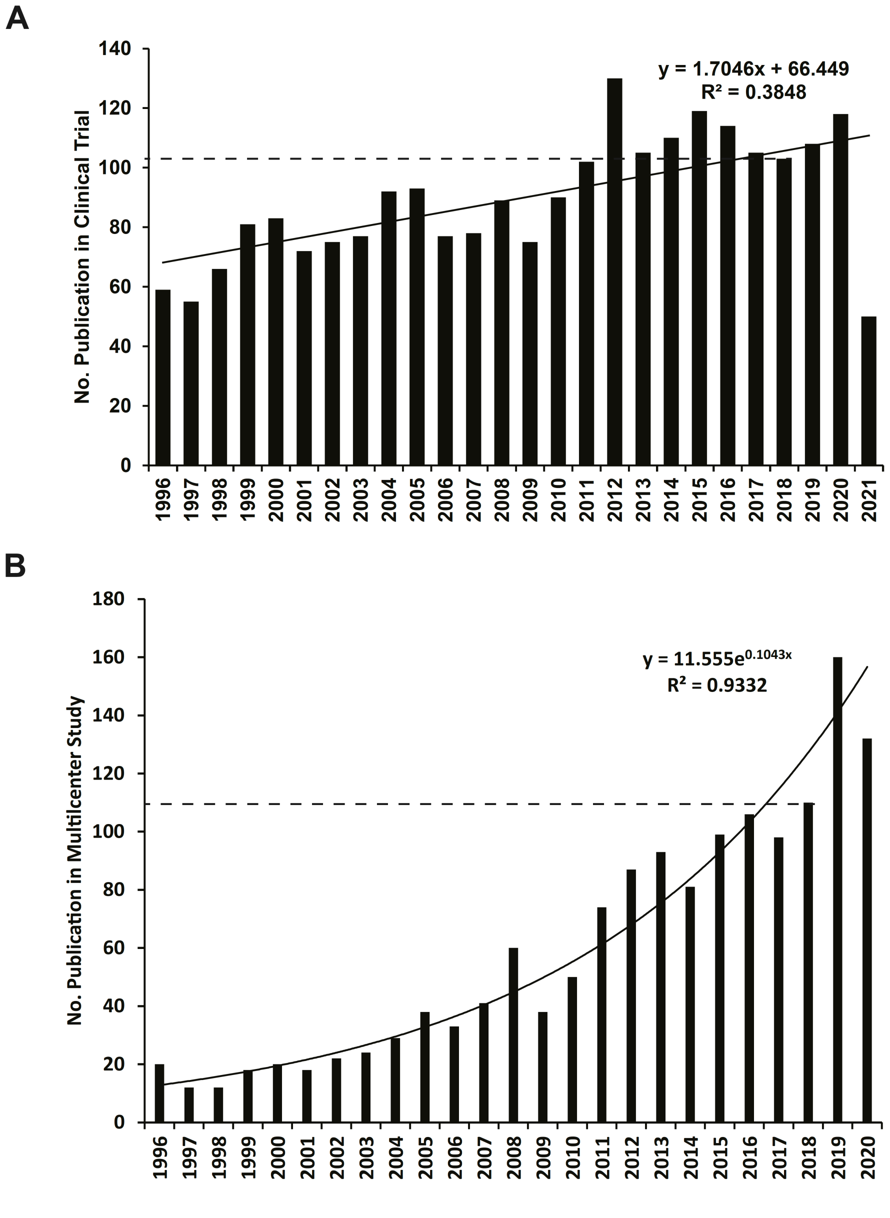

Figure S1. The number of publications in the fields of "Clinical Trials" and "Multicenter Studies" increased over the years. (A) According to the inclusion criteria, 2,326 publications were included in the analysis. Excluding the incomplete data of 2021, an average of 90 clinical trials are published every year. The linear growth function is y=1.7046x+66.449, R^2 is 0.38, and the growth is relatively stable, so it is predicted that there will be 110-115 in 2021 and 2022. (B) The field of "Multicenter Study" has an average of 59 publications published each year, showing an exponential growth function. The proper function of the number of publications is $y=11.55e^{0.10433x}$, and R^2 is 0.9332. It is predicted that there will be 173 and 193 publications in 2021 and 2022. The dotted line marks the number of publications in 2018.

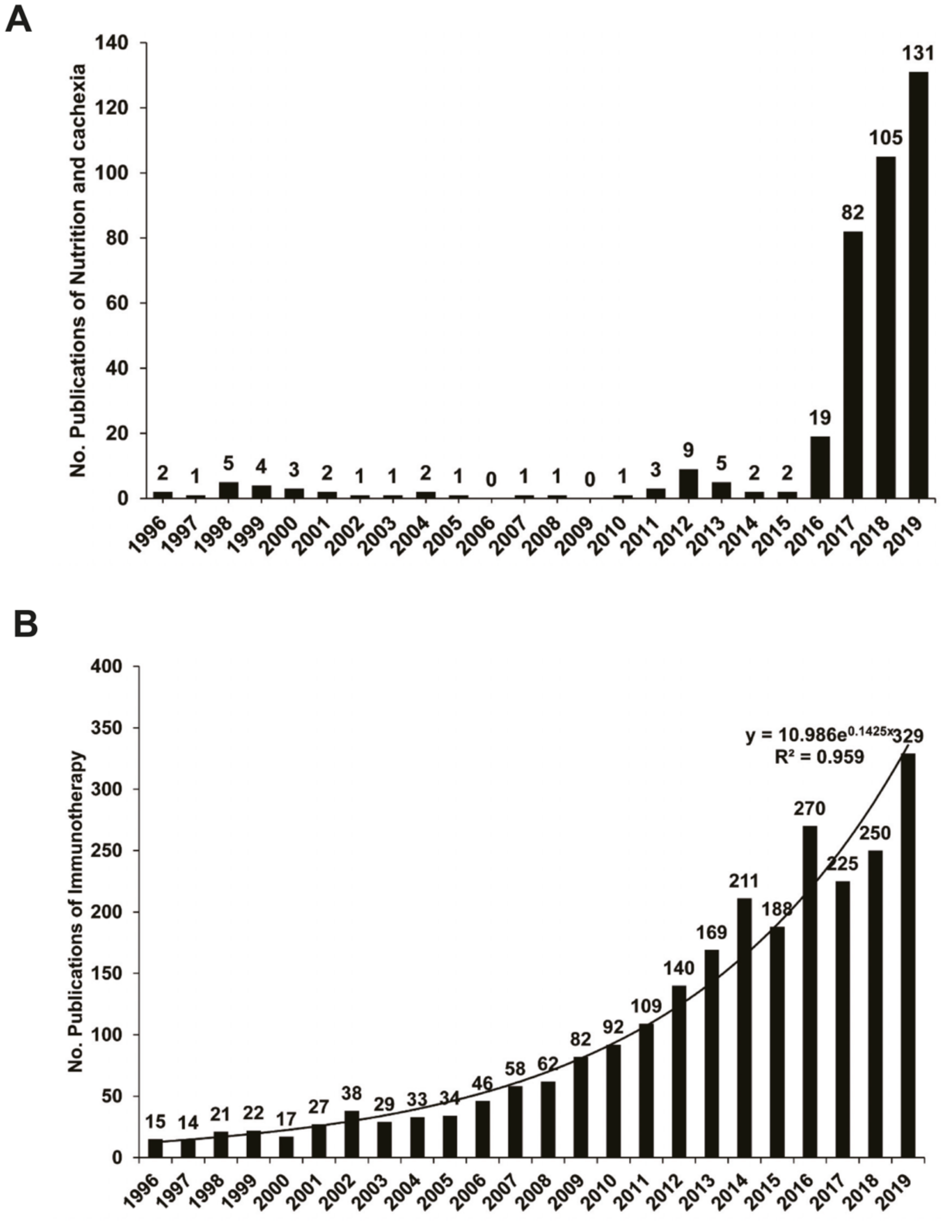

Figure S2. The number of publications in the fields of "Nutrition and cachexia" and "Immunotherapy" increased. For description, see legend of Figure S1.

Supplemental information 1 Core code of LDA methods used. The searching and downloading of the publications were carried out through the R package easyPubMed, all exclusions were carried out through manual evaluation, the LDA analysis was carried out through Python, and the visualization was made with Excle and R.

more information can be found in GitHub (https://github.com/yanwen0614/Medicine-Bibliometric-Analysis).

```
from nltk.tokenize import RegexpTokenizer
#from stop_words import get_stop_words
from nltk.stem.porter import PorterStemmer
from sklearn.utils import shuffle
from gensim import corpora, models
import pandas as pd
import logging
import pickle
import numpy as np
import os,sys
logging.basicConfig(level = logging.INFO,format = '%(asctime)s - %(name)s - %(levelname)s - %(message)s')
logger = logging.getLogger(__name__)

from gensim.models.ldamulticore import LdaMulticore

def dumppick(filepath,Year):
    corpus = []     # Store documents
    tokens = []     # Store the words in the document

    df = pd.read_csv(filepath,sep='\t',encoding="utf-8-sig",error_bad_lines=False)
    df = df[df["Abstract"].isna()!=True]
    df.astype({'year': 'int32'})
    df = df[df.year<Year]
    # Operation of reading the document
    for line in df["Abstract"]:
        corpus.append(line.strip())
    del df
    # Remove punctuation and stop words
    en_stop = [ str(i).strip() for i in open("stopwords.txt",encoding="utf-8-sig") ]

    # extracting the main words
    p_stemmer = PorterStemmer()

    logging.info("WBYCL")
```

```
tokenizer = RegexpTokenizer(r'[A-Za-z]+')
for i,text in enumerate(corpus):
    if i%1000==0:
        logging.info(f"{i} line done")
    raw = text.lower()
    token = tokenizer.tokenize(raw)
    stop_remove_token = [word for word in token if (word not in en_stop and
len(word)>1)]
    stem_token = [p_stemmer.stem(word) for word in stop_remove_token]
    tokens.append(stem_token)
    # tokens.append(token)
# print tokens

logging.info("Start to calculate the document-word matrix (directly use the
statistical word frequency to get the features) ")


logging.info("Bscia calculationn")

dictionary = corpora.Dictionary(tokens)
# print dictionary.token2id
# print type(dictionary)
logging.info("Document-word matrix for bag of words model ")
texts = [dictionary.doc2bow(text) for text in tokens]
logging.info("Calculation complete ")


logging.info("start tfidf")
texts_tf_idf = models.TfidfModel(texts)[texts]
# # for text in texts_tf_idf:
# #     print text
# lda_tf_idf = models.LdaModel(texts_tf_idf, num_topics=3, id2word=dictionary,
update_every=0, passes=200)
# print lda_tf_idf.print_topics(num_topics=3,num_words=4)
# # doc_topic = [a for a in lda_tf_idf[texts_tf_idf]]
# # for topic_id in range(3):
# #     print "topic:{}".format(topic_id+1)
# #     print lda_tf_idf.show_topic(topic_id)
# corpus_lda_tfidf = lda_tf_idf[texts_tf_idf]
# for doc in corpus_lda_tfidf:
#     print doc
pickle.dump(texts, open("text_dtm.pickle","wb"))
pickle.dump(texts_tf_idf, open("texts_tf_idf_dtm.pickle","wb"))
pickle.dump(dictionary, open("dictionary.pickle","wb"))
```

```
def loadpcik():
	texts = pickle.load(open("text_dtm.pickle","rb"))
	texts_tf_idf = pickle.load(open("texts_tf_idf_dtm.pickle","rb"))
	dictionary = pickle.load(open("dictionary.pickle","rb"))
	return texts, texts_tf_idf,dictionary

#dumppick()
def createlda(num_topics,filename,Year):
	dumppick(filename,Year)
	texts, texts_tf_idf, dictionary = loadpcik()

	"""
	print("**************LSI*************")
	lsi = models.lsimodel.LsiModel(corpus=texts, id2word=dictionary, num_topics=20)
	texts_lsi = lsi[texts_tf_idf]
	print(lsi.print_topics(num_topics=20, num_words=10))
	"""

	logging.info("**************LDA*************")

	lda = LdaMulticore(corpus=texts,iterations=1000, id2word=dictionary, num_topics=num_topics,passes=300,per_word_topics=True)
	#texts_lda = lda[texts_tf_idf]
	out = open("./ldamd/{}tpc-tpc".format(num_topics),mode="w",encoding ="utf-8-sig")
	# print(lda.print_topics(num_topics=num_topics, num_words=10),file =out)
	lda.save("./ldamd/{}tpc+{}".format(num_topics,filename[9:18]))
		#ppl.append(np.exp2(-lda.log_perplexity(texts_vad))/i)
	return lda,texts, texts_tf_idf, dictionary

def lodaldaml():
	lda = models.LdaModel.load("./ldamd/50tpc")

def saveldatpcw(lda):
	tpcn = 50
	tpcw = pd.DataFrame(columns=[i for i in range(1,11)])
	for i in range(tpcn):
		tpcw.loc[i] = [ w for w,p in lda.show_topic(i)]
	tpcw.to_csv("./newdata/tpcw.csv")


def get_cite_n_dmt(dictionary,citenum=0,):
```

```
        citenum=0
        corpus = []
        tokens = []
        df = pd.read_csv("pubmed_result_parsed.csv",sep=',',encoding="utf-8-sig")
        df = df[df["References"]==citenum]
        df = df[df["Abstract"].isna()!=True]
        for line in df["Abstract"]:
                corpus.append(line.strip())
        del df
        en_stop = [ str(i).strip() for i in open("stopwords.txt",encoding="utf-8-sig") ]

        p_stemmer = PorterStemmer()

        logging.info("WBYCL")
        tokenizer = RegexpTokenizer(r'[A-Za-z]+')
        for i,text in enumerate(corpus):
                if i%1000==0:
                        print(i)
                raw = text.lower()
                token = tokenizer.tokenize(raw)
                stop_remove_token = [word for word in token if (word not in en_stop and
len(word)>1)]
                stem_token = [p_stemmer.stem(word) for word in stop_remove_token]
                tokens.append(stop_remove_token)
        texts_cite_n = [dictionary.doc2bow(text) for text in tokens]
        return texts_cite_n

def get0cited2tpc(lda,texts):
        tpc1 = []
        tpc2 = []
        for i in texts_cite_0:
                tpc = lda.get_document_topics(i)
                tpc = sorted(tpc,key=lambda x:-x[1])
                tpc1.append(tpc[0][0])
                if len(tpc)>1:
                        tpc2.append(tpc[1][0])
                else:
                        tpc2.append(lda.num_topics+1)
        df = pd.read_csv("pubmed_result_parsed.csv",sep=',',encoding="utf-8-sig")
        df = df[df["Refecrence"]==0]
        df = df[df["Abstract"].isna()!=True]
        df["tpc1"] = tpc1
        df["tpc2"] = tpc2
        df.to_csv("0cite_tpc.csv")
```

```
    return tpc1,tpc2

def getallcited2tpc(lda,texts,filename,Year):
    tpc1 = []
    tpc2 = []
    for i in texts:
        tpc = lda.get_document_topics(i)
        tpc = sorted(tpc,key=lambda x:-x[1])
        tpc1.append(tpc[0][0])
        if len(tpc)>1:
            tpc2.append(tpc[1][0])
        else:
            tpc2.append(lda.num_topics+1)
    df = pd.read_csv(filename,sep='\t',encoding="utf-8-sig")
    df = df[df["Abstract"].isna()!=True]
    df = df[df.year<Year]
    df["tpc1"] = tpc1
    df["tpc2"] = tpc2
    df.to_csv(filename.replace(".csv","_with_topic.csv"),sep='\t',encoding="utf-8-
sig")
    return tpc1,tpc2

def grap(tpc1,tpc2,tpcn,filename):
    from collections import  Counter
    CC = Counter(tpc1)
    import networkx as nx
    G = nx.Graph()
    for i in range(tpcn):
        G.add_node(i,num=CC[i])
    #edgscount = Counter([(i,j) for i,j in zip(tpc1,tpc2)])
    #for edgs,count in edgscount.items()
    edgeslist = [(i,j)    for i,j in zip(tpc1,tpc2) if j<tpcn]
    G.add_edges_from(edgeslist,Weight=0)
    for i,j in zip(tpc1,tpc2):
        if j >= tpcn:
            continue
        G.edges[i,j]["Weight"]+=1
    nx.write_graphml(G,filename.replace(".csv",".graphml"),encoding="utf8")
    return G

def Grap_Add_tpcname():
    import pandas as pd
    c = pd.read_excel("glioblastoma\\Theme naming-gbm.xlsx")
    pic = {tpcid:name for tpcid,name in  zip( range(50),c["Theme naming"])}
```

```
    import networkx as nx
    g = nx.read_graphml("glioblastoma\\Glioblastoma_50.graphml")
    for i in range(50):
        g.node[str(i)]["name"] = pic[i]

nx.write_graphml(g,"glioblastoma\\Glioblastoma_50.graphml_addname.graphml",enc
oding="utf8")

def main(Year):
    filename = "total.csv"

    if not os.path.exists("./newdata"):
        os.mkdir("./newdata")
    if not os.path.exists("./ldamd"):
        os.mkdir("./ldamd")

    lda,texts, texts_tf_idf, dictionary    = createlda(50,filename,Year)

    saveldatpcw(lda)
    logging.info("LDA Finished")
    logging.info("The subject of each article is being calculated ")
    tpc1,tpc2 = getallcited2tpc(lda,texts,filename,Year)

    logging.info("Making a connection diagram")
    grap(tpc1,tpc2,50,filename)

if __name__ == "__main__":
    Year = 2020
    main(Year)
```
